# Inhalation delivery of topotecan is superior to intravenous exposure for suppressing lung cancer in a preclinical model

**DOI:** 10.1080/10717544.2018.1469688

**Published:** 2018-05-19

**Authors:** Philip J. Kuehl, Marcie J. Grimes, Devon Dubose, Michael Burke, David A. Revelli, Andrew P. Gigliotti, Steven A. Belinsky, Mathewos Tessema

**Affiliations:** a Lovelace Biomedical, Albuquerque, NM, USA;; b Lung Cancer Program, Lovelace Respiratory Research Institute, Albuquerque, NM, USA;; c Lonza-Bend Research Institute, Bend, OR, USA

**Keywords:** Topotecan, inhalation, lung cancer, aerosol, dry powder

## Abstract

Intravenous (IV) topotecan is approved for the treatment of various malignancies including lung cancer but its clinical use is greatly undermined by severe hematopoietic toxicity. We hypothesized that inhalation delivery of topotecan would increase local exposure and efficacy against lung cancer while reducing systemic exposure and toxicity. These hypotheses were tested in a preclinical setting using a novel inhalable formulation of topotecan against the standard IV dose. Respirable dry-powder of topotecan was manufactured through spray-drying technology and the pharmacokinetics of 0.14 and 0.79 mg/kg inhalation doses were compared with 0.7 mg/kg IV dose. The efficacy of four weekly treatments with 1 mg/kg inhaled vs. 2 mg/kg IV topotecan were compared to untreated control using an established orthotopic lung cancer model for a fast (H1975) and moderately growing (A549) human lung tumors in the nude rat. Inhalation delivery increased topotecan exposure of lung tissue by approximately 30-fold, lung and plasma half-life by 5- and 4-folds, respectively, and reduced the maximum plasma concentration by 2-fold than the comparable IV dose. Inhaled topotecan improved the survival of rats with the fast-growing lung tumors from 7 to 80% and reduced the tumor burden of the moderately-growing lung tumors over 5- and 10-folds, respectively, than the 2-times higher IV topotecan and untreated control (*p* < .00001). These results indicate that inhalation delivery increases topotecan exposure of lung tissue and improves its efficacy against lung cancer while also lowering the effective dose and maximum systemic concentration that is responsible for its dose-limiting toxicity.

## 1. Introduction

Lung cancer is the leading cause of cancer-related mortality in the US and worldwide (Torre et al., 2015; Siegel et al., 2017). The high incidence rate of lung cancer combined with limited and ineffective treatment options available for most patients with advanced disease have led to this deadliest ranking. Curative intent resection remains the gold standard and the most effective treatment option. Unfortunately, more than two-thirds of lung cancer patients do not qualify for surgery due to advanced stage disease or other health issues. Although recent progresses in targeted- and immuno-therapy are showing encouraging results in a small subset of patients whose tumors show specific sensitivity markers, nearly all targeted therapy responders develop resistance within a year or two (Pao & Girard, 2011; Meng et al., 2017). The limitation of immunotherapy even among lung cancer patients that are positive for the sensitivity marker (PD-L1) is also demonstrated in the recently published results from the CheckMate clinical trial (Carbone et al., 2017). This open-label phase-III study revealed that the efficacy of nivolumab for the treatment of PD-L1 positive stage IV non-small cell lung cancer (NSCLC) patients in a first-line setting was similar with platinum-based chemotherapy (Carbone et al., 2017). Thus, the majority of lung cancer patients still rely on chemotherapy but less than 5% survive for 5-years due to the low efficacy and strong side effects of currently used drugs. As the effectiveness of traditional chemotherapy has plateaued over the last decade, new approaches are urgently needed. Although the discovery and development of anticancer agents is an area of intense research, it is an extremely lengthy and costly process with high failure rates. Furthermore, the clinical use of some cancer drugs that survive the developmental and approval processes is also undermined by severe toxicity in some patients. Thus, improving or re-purposing approved but rarely used potent anticancer drugs such as topotecan that will allow shorter development process and faster path to clinical use due to well-defined toxicity profile in patients is an attractive approach.

Topotecan (US Brand Name Hycamtin) is a topoisomerase-I inhibitor that is approved for the treatment of various solid tumors including small cell lung cancer and is widely available in the clinic. The US Food and Drug Administration (FDA) approved topotecan intravenous (IV) infusion for multiple cancer types in 1996 and its oral capsule formulation was approved in 2007 (O’Brien et al., 2006; Eckardt et al., 2007; O’Brien et al., 2007). Clinical trials using topotecan as a single-agent or in combination with other drugs have also demonstrated efficacy for NSCLC (Lynch et al., 1994; Perez-Soler et al., 1996; Kindler et al., 1998; Weitz et al., 2000; Ramlau et al., 2006; Jones et al., 2008). The anti-tumor activity of topotecan in advanced NSCLC patients has been shown to be comparable to the standard second-line drugs for this disease such as paclitaxel and docetaxel (Ramlau et al., 2006; Jones et al., 2008). However, the clinical use of topotecan is greatly restricted and its efficacy undermined by severe dose-limiting hematological toxicity in some patients (O’Brien et al., 2006; Eckardt et al., 2007; O’Brien et al., 2007). Although the major hematopoietic tissues that are highly sensitive to topotecan are often located far away from the solid tumor target (e.g. lung tumors), they are exposed to higher concentration of the drug delivered through IV route. The anticancer efficacy of topotecan is also significantly undermined by the systemic delivery because the maximum tolerated dose (MTD) and the amount of drug reaching the cancer cells that are often located deep in remote tissues is lower than the optimum concentration needed for anticancer activity.

In this study, we aimed to improve the efficacy and expand the utility of topotecan for lung cancer therapy through direct inhalation delivery that will reverse the two major disadvantages of IV topotecan, high systemic and low lung tumor exposures. We hypothesized that targeted delivery of topotecan into the lungs through inhalation will increase the drug exposure of lung tumors by achieving higher maximum concentration (*C*_max_) and area under the curve (AUC) of lung tissue and thereby significantly increases its anticancer efficacy. Similarly, retention of most of the inhaled drug in the lungs and its slower release from the lungs to the systemic circulation will reduce the systemic *C*_max_, minimize exposure and toxicity to the hematopoietic tissue, and thereby lower the major dose-limiting toxicity that is greatly undermining the clinical use of this potent and widely available anticancer agent.

The major goals of this study were to develop an inhalable dry-powder formulation of topotecan and characterize its aerosol properties, pharmacokinetics, and pre-clinical efficacy against lung cancer in an orthotopic model. The effects of the treatments on survival, lung tumor burden, and potential hematopoietic and other toxicities were evaluated.

## 2. Materials and methods

### 2.1. Manufacturing and in vitro characterization of spray-dried powder of topotecan

A 100% aqueous solution of topotecan with 3% total solids by weight was prepared from (S)-Topotecan (Toronto Research Chemicals Inc., Toronto, Canada). The pH was adjusted to 3.5 with 1 M HCl to ensure complete dissolution and the excipients Trehalose and L-leucine were added for stability and particle formation. The solution was spray-dried using a two-fluid atomization nozzle with a lab-scale custom spray dryer (BLD-35). Conditions were set to; 500 g/min drying gas flow rate, 8 ml/min liquid feed rate, 45 psi atomization pressure, and 60 °C outlet temperature. The analytical tests conducted to determine the physical, chemical, and aerosol characteristics of the dry powder formulation included; water content by Karl Fischer, particle size distribution (PSD) by Malvern Mastersizer wet-method, particle morphology by scanning electron microscope (SEM), physical state by X-ray powder diffraction (XRPD), thermal properties by modulated differential scanning calorimetry (mDSC), and potency/purity by RP-HPLC. The aerosol properties of the spray-dried topotecan powder including the mean mass aerodynamic diameter (MMAD), fine particle fraction (FPF), emitted fraction (EF), geometric standard deviation (GSD), and powder distribution size plots were assessed using an RS01 capsule based device and a Next Generation Impactor (NGI model 170, MSP Corp.) operated at 60 l/min, 4 l total air flow, and data from replicates were analyzed using CITDAS version 3.10 (Copley Scientific Ltd., Nottingham, UK).

### 2.2. *In vivo* delivery of topotecan

All *in vivo* procedures were conducted at Lovelace Biomedical under protocols approved by the Lovelace Institutional Animal Care and Use Committee. Lovelace facilities are accredited by the Association for Assessment and Accreditation of Laboratory Animal Care (AAALAC) International. The aqueous formulation of topotecan for IV delivery was prepared immediately prior to injection according to the recommendation for HYCAMTIN^®^ (topotecan) for injection. A 1 mg/ml topotecan solution for injection was prepared under sterile condition using 5% dextrose and the dose volume for each animal was adjusted based on body weights and injected through the tail vein. The inhalation doses were given using a rodent nose only inhalation exposure system (supplementary figure, Figure S1) in which, the spray-dried topotecan powder aerosols were generated by a rotating brush generator and dose adjustments were made by modulating the aerosol concentration and the duration of exposure. Pulmonary-deposited doses were calculated with standard methods utilizing a deposition fraction of 10% (Alexander et al., 2008). The exposure system was developed and characterized over the dose range(s) required prior to exposures for concentration and PSD. Aerosols were monitored for total aerosol concentration, topotecan aerosol concentration, and PSD at the breathing zone of the exposure system.

### 2.3. Pharmacokinetic analysis of inhaled versus intravenous topotecan

The pharmacokinetics of 0.7 mg/kg IV topotecan was compared with two different doses of inhaled topotecan (0.14 and 0.79 mg/kg) using the Sprague Dawley rats. The IV and inhalation doses were delivered to a total of 90 rats (30 rats per dose) as described above and 3 rats were serially sacrificed from each dose group at 9 time-points over 24 h (5, 15, and 30 min, 1, 2, 4, 8, 12, and 24 h). At each time-point, systemic blood was collected into K_3_EDTA tubes, the plasma separated, and stored at −80 °C until analysis *via* the LCMS assay while lung tissue was snap frozen on liquid nitrogen. The plasma samples were prepared *via* a protein precipitation method with 1% formic acid in acetonitrile. The lung samples were first homogenized at a ratio of 1 part lung tissue to 4 parts organic (1% formic acid in acetonitrile) and then underwent the same protein precipitation as the plasma samples prior to LCMS analysis. Camptothecin was used as the internal standard in both lung and plasma samples. Separation was performed with a Waters H-Class UPLC on a Zorbax C_8_ column (2.1 × 50 mm, 3.5 µm) with a ballistic gradient of 0.15 formic acid in water and 0.1% formic acid in acetonitrile over 2.5 min. Quantification was performed in MRM on an ABSciex API 4000 based on matrix based standards between 5 and 5000 ng/ml for plasma and 20 and 20,000 ng/ml in lung tissue. Linear regress was performed with 1/*x*^2^ weighting for both matrices. Standard bioanalytical matrix based run quality check’s (QC’s) were also included.

### 2.4. Efficacy of inhaled versus IV topotecan for lung cancer therapy

The efficacy of inhaled topotecan to treat lung cancer was compared to the standard IV administration using an orthotopic lung cancer model that is well-established in our lab (March et al., 2001; Belinsky et al., 2011; Reed et al., 2013). A total of 96 male Rowett nude rats (Cr:NIH-rnu) and two human lung adenocarcinoma cells lines (A549 and H1975) were used to generate two types of lung tumors. The two cell lines were obtained from American Type Culture Collection (ATCC), maintained according to ATCC protocols, and used within 6 months post-resuscitation. One day prior to implantation of the cancer cells (day-1), the nude rats received whole-body X-irradiation to augment immunosuppression and improve engraftment of lung tumors. All animals were weighed to obtain the pre-study body weight and randomized into treatment groups as shown in Table 1. Group 1 animals were excluded from tumor implantation and treatment to serve as normal (cancer-free and treatment-free) control. On day 0, A549 and H1975 cell lines (15 × 10^6^ cells/rat) were instilled *via* the trachea into the lungs of 45 rats each in Groups 2–4 and 5–7, respectively. All animals were weighed once weekly for the duration of the study. Starting on day 25, after three weeks of tumor establishment and growth, the rats were treated once-a-week for 4 consecutive weeks with filtered air (Groups 2 and 5), 2 mg/kg IV topotecan *via* tail vein injections (Groups 3 and 6), or 1 mg/kg topotecan *via* inhalation (Groups 4 and 7). The weekly 2 mg/kg IV dose was scaled based on the clinical dose used for small-cell lung cancer patients, 1.5 mg/m^2^/d for 5 d (Eckardt et al., 2007; von Pawel et al., 2014) or 4–6 mg/m^2^ weekly (Masuda et al., 2010; Allen et al., 2014). Based on the PK data that suggested better efficacy of inhaled topotecan, 1 mg/kg (half of the IV dose) was used for inhalation delivery. Animals were sacrificed for moribund conditions or at the end of the study on day 54 post-tumor implantation.

**Table 1.** Experimental design of inhaled vs. IV topotecan for the treatment of lung tumors in rats.

Group	Lung cancer Cell line	Number of rats	Treatments**		
			Vehicle	2 mg/kg IV	1 mg/kg inhalation
1	None*	6	−	−	−
2	A549	15	+	−	−
3		15	−	+	−
4		15	−	−	+
5	H1975	15	+	−	−
6		15	−	+	−
7		15	−	−	+

* Animals in Group 1 serve as cancer-free and treatment-free control.

** The ‘+’ and ‘−’ signs indicate the treatments given or not given, respectively.

### 2.5. Terminal tissue collection and measurements of tumor burden

Terminal blood samples were collected through cardiac puncture, blood smears were prepared, and complete and differential blood counts were done using the Siemens Advia™ 120 hematology analyzer. The lungs from all animals were excised and weighed with tracheas attached. Additional samples including bone marrow, spleen, gastrointestinal tract (GIT) and lung tumors (when present) were collected from randomly pre-selected half of the animals in each group. Lung tumors (5 per animal) were collected from the preselected animals in 10% neutral-buffered formalin (NBF) for histology and also in screw cap tubes and snap frozen in liquid nitrogen for future molecular analysis. The remaining lungs were inflated with 10% NBF at a constant hydrostatic pressure of 25 cm for 6 h. Bone marrow smears were prepared from the femur immediately after collection and the remaining bone marrow and other tissues were placed in 10% NBF for histology. Paraffin-embedded tissues were prepared, sectioned at 5 μm thickness, and stained with hematoxylin and eosin (H&E).

### 2.6. Statistical analyses

Pharmacokinetic analysis was performed with WinNonlin version 6.2 (Pharsight Corp., Sunnyvale, CA) using non-compartmental analysis of average concentrations of topotecan in plasma and lung samples that were above the lower quantitation limit. The half-life (T_1/2_), time to maximum concentration (*T*_max_), maximum concentration (*C*_max_), AUC, and dose-normalized AUC (AUC_dose_) of the drug were determined following IV or inhalation delivery. Tumor burden was quantified based on the changes in lung weight of a tumor-bearing lung compared to the average weight of the normal lungs from the age-matched Group-1 animals. Our previous studies demonstrated that treatment-related reduction in tumor burden was highly correlated with estimates of tumor volume (Belinsky et al., 2011; Reed et al., 2013). The two-sample t-test and analysis of variance were used to compare the two treatment groups and each treatment group with the vehicle, respectively. To check the appropriateness of the statistical methods, analyses were re-run with transformed data if appropriate or using the Kruskal–Wallis test.

## 3. Results

### 3.1. Manufacturing of a stable inhalable topotecan

**Figure 1.**
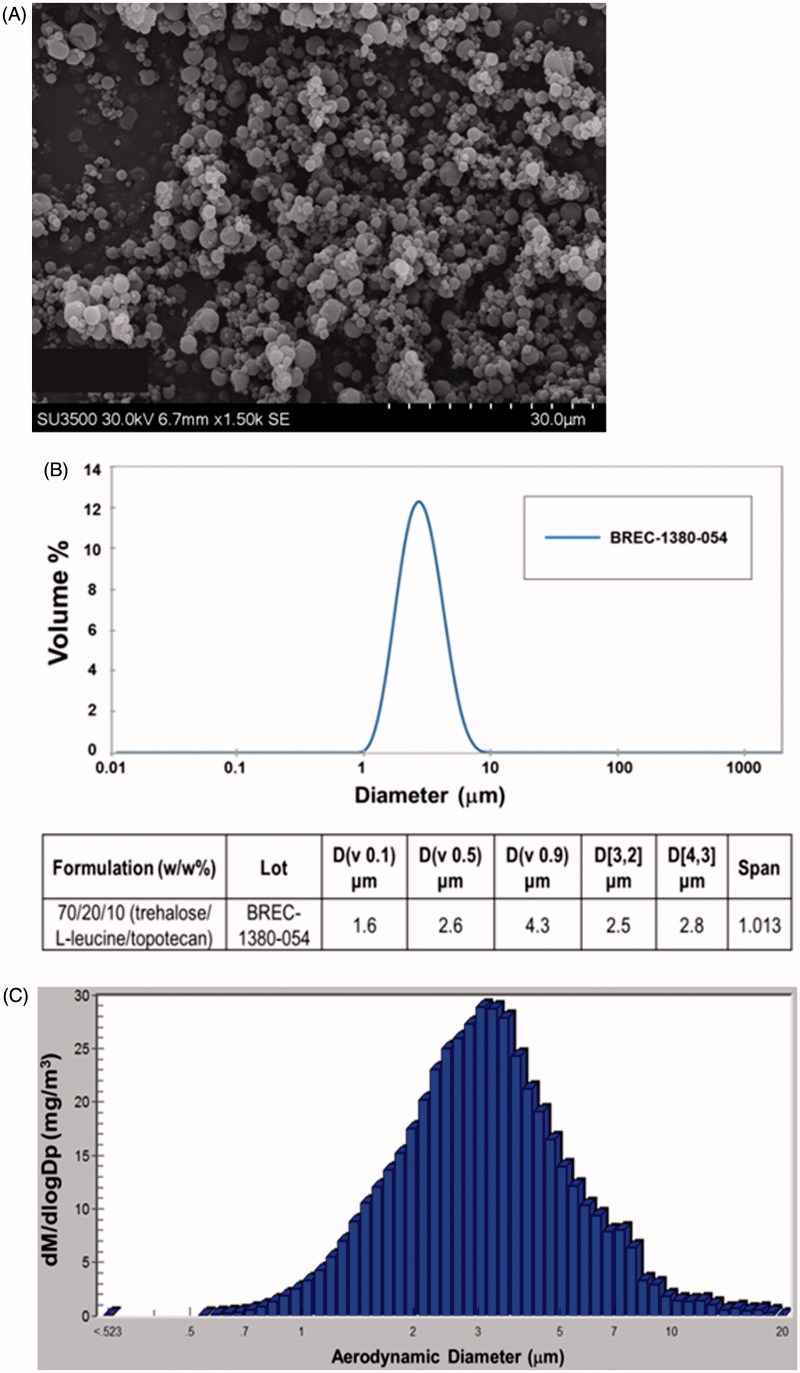
Physical properties of the spray-dried powder of topotecan. (A) Scanning Electron Microscope image. (B) Volume particle size distribution (Malvern–Wet Method) and (C) mass particle size distribution recovered from the inhalation chamber.

A respirable dry-powder that contains topotecan as the active pharmaceutical ingredient (API) along with the standard excipients Trehalose and L-leucine was produced by Lonza-Bend Research Institute using a lab-scale spray-drying. The product is composed of 70/20/10 (w/w%) trehalose/L-leucine/topotecan and resulted in 92% yield. Evaluation of the physical and chemical characteristics and PSD of the formulation revealed that the dry-powder contained 2.92% residual water by weight, has rigid fusion-free particle morphology, and a homogeneous PSD of <10 µm (Figure 1(A–C)). The purity of topotecan in the post-spray-dried powder was analyzed by HPLC with an extended assay (60 min injection run time) and was found to be over 96% pure. Physical state analysis of the product using XRPD revealed that it contains crystalline leucine while trehalose and topotecan have amorphous compositions. Analysis of the product by mDSC indicated a glass transition (Tg) temperature of 117 °C when held dry and 62 °C at 20% relative humidity that indicate the product is stable when held dry at controlled ambient temperature. The topotecan powder contained a 97% theoretical potency while only having 0.23% more impurities than the reference standard (not shown). The aerosol property of the dry powder was evaluated *in vitro* using Size 3 HPMC capsule and actuated into a NGI using a Plastiape RS01 High Resistance device. The emitted fractions (EF) from the capsule and device on average was 94% and the corresponding MMAD, GSD, and FPF were 2.9 µm, 1.9 µm, and 62%, respectively (Table S1). These results indicate a highly respirable, dry powder with excellent physical characteristics to achieve reduced oral and increased pulmonary deposition that is optimum for *in vivo* assessment.

### 3.2. Pharmacokinetic analysis of inhaled topotecan

**Figure 2.**
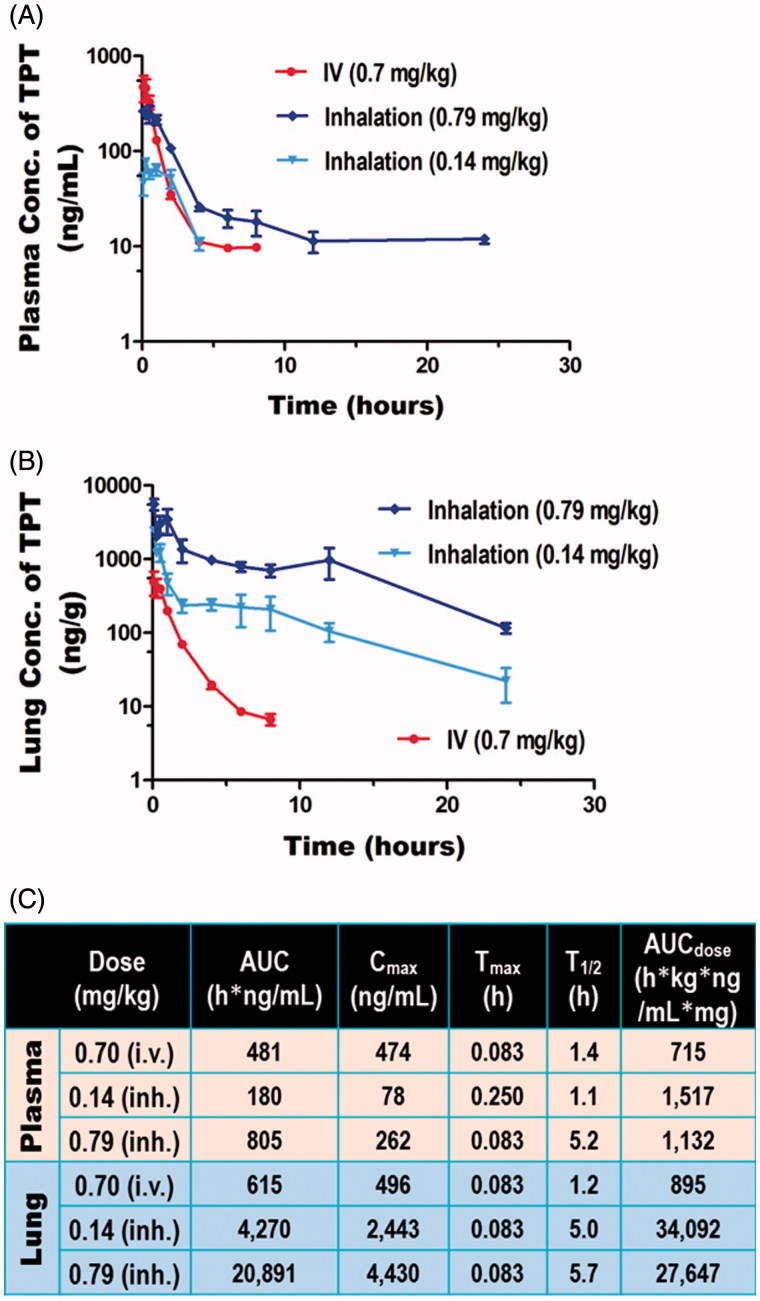
Pharmacokinetic Analysis of Inhaled versus IV topotecan. The levels of topotecan detected in (A) plasma and (B) lung tissue, and (C) the average values from non-compartmental analysis of concentration vs. time are shown.

The pharmacokinetics of two different doses of inhaled topotecan (0.14 and 0.79 mg/kg) was compared with 0.70 mg/kg IV topotecan using Sprague Dawley rats. The inhalation doses were given using a rodent nose only inhalation exposure system (Figure S1) while the IV dose was given *via* tail vein injection. The actual aerosol concentration, particle size, and pulmonary deposited dose(s) are shown in Table S2 and a histogram depicting the PSD of the drug recovered from the inhalation chamber is shown in Figure 1(C). The results showed that, over the dose range tested and the delivery routes evaluated, topotecan undergoes apparent first-order elimination. The concentration vs. time profile showed a bi-phasic elimination profile from the plasma for both inhaled and IV topotecan (Figure 2(A)). The clearance from systemic circulation and lung following inhalation delivery of 0.79 mg/kg topotecan was 3.7- and 4.8-times slower [T_1/2_ (h.) = 5.2 vs. 1.4 and 5.7 vs. 1.2], respectively, than the 0.70 mg/kg IV dose. Similarly, inhalation delivery of 0.79 mg/kg topotecan resulted in 34-fold higher exposure of the lung tissue compared to IV delivery of 0.70 mg/kg, AUC (h*ng/ml) = 20,891 vs. 615 (Figure 2(A–C)). Similarly, the use of 5-times lower dose of topotecan *via* inhalation than IV delivery (0.14 vs. 0.70 mg/kg) resulted in 4.2-times longer half-life in the lungs (5.0 vs. 1.2 h.) and 7-fold higher lung tissue exposure, AUC = 4270 vs. 615 h*ng/ml (Figure 2(C)).

### 3.3. Effects of inhaled topotecan on lung cancer survival

The efficacy of inhaled topotecan to treat lung cancer was compared with the standard IV administration using a well-established orthotopic lung cancer model in rats. Two human lung adenocarcinoma cell lines, H1975 and A549, were respectively used to generate aggressive fast-growing and moderately-growing tumors in the lungs of the nude rat as described (March et al., 2001; Belinsky et al., 2011; Reed et al., 2013). Each cell line was instilled into the lungs of 45 rats that were divided into three treatment groups as shown in Table 1. Six extra rats (Group 1) were kept as tumor and treatment naïve normal control. After three weeks of tumor growth, the rats were treated once-a-week for 4 weeks with vehicle (filtered air and supportive care as necessary), 2 mg/kg IV topotecan, or 1 mg/kg topotecan aerosol *via* inhalation. The average pulmonary-deposited dose and PSD of the inhalation dose used for the efficacy study was found to be 0.99 mg/kg and 2.9 µm, respectively (Table S2).

**Figure 3.**
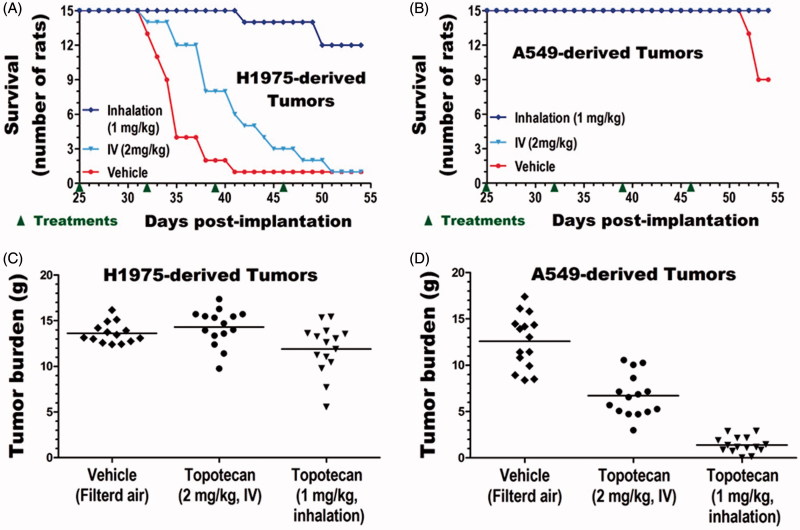
Efficacy of inhaled versus IV topotecan for lung cancer therapy. The survival (A,B) and lung tumor burden (C,D) of rats with the fast-growing H1975 (A and C) and moderately growing A549 (B and D) derived lung cancer were compared between treatment groups.

The impacts of treatments on survival were compared for each tumor type. The end of study (54 d post tumor implantation) survival rate of untreated cancer-free control animals (6/6) and IV (15/15) or inhaled (15/15) topotecan treated animals with A549-derived lung tumors was 100% (Table S3). In contrast, the survival rates of animals with untreated A549-derived tumors (9/15), untreated H1975-derived tumors (1/15), and IV (1/15) or inhaled (12/15) topotecan treated animals with H1975-derived tumors were 60, 7, 7, and 80%, respectively (Figure 3(A–B), Table S3). These results indicate that inhaled topotecan dramatically improved the survival of rats with the aggressive H1975-derived lung tumors from just 7% in the 2-times higher IV dose to 80% (*p* < .0001, Figure 3(A)). The end of study survival rate of IV topotecan treated animals with this aggressive lung cancer was as low as the untreated control (both 7%). However, survival of only 13% (2/15) of untreated control animals on day 40 compared to 53% (8/15) of IV topotecan and 100% (15/15) of inhaled topotecan treated rats indicates that IV topotecan also had some minor effect on this tumor type (Figure 3(A)). In contrast, the end of study mortality rate of the moderately growing A549-derived lung cancer was low. Thus, survival analysis of this less aggressive tumor type did not discriminate the impacts of inhaled vs. IV topotecan, but showed that both treatments improved survival from 60% in untreated control to 100% (Figure 3(B), Table S3).

### 3.4. Effects of inhaled topotecan on lung tumor burden

The lungs of all animals were collected immediately after sacrifice or death and weighed. The normal lung weight for age-matched, untreated, and cancer-free rats was obtained from Group 1 animals (*n* = 6). The lung tumor burden of each tumor-bearing animal in Groups 2–7 (*n* = 15 per group) was determined by subtracting the average lung weight of the six normal lungs from the weight of each tumor-bearing lung. The mean and standard deviation of the lung weights for each treatment group as well as the tumor burdens of each tumor-bearing group are shown in Table 2. The average lung tumor burden of untreated, IV, or inhaled topotecan treated rats with H1975-derived tumors was 14.3, 15.8, and 13.4 g, respectively, and was not significantly different between the three treatment groups (Figure 3(C)). However, as tumors in the different treatment groups grew for quite different time period, the terminal tumor burden of this aggressive tumor type could not accurately estimate the rate of tumor growth. In contrast, all IV or inhaled topotecan treated and most untreated animals with the less aggressive A549-derived tumors survived to the end of the study (Figure 3(B) and Table S3). Hence, the impacts of the different treatments on tumor growth could be more accurately determined from the terminal tumor burden of this tumor type. Both IV and inhaled topotecan treatments significantly reduced the lung tumor burden of A549-derived tumors compared to the untreated control (*p* < .00001, Figure 3(D)). Specifically, IV and inhaled topotecan respectively reduced the tumor burden by 1.9-fold (47%) and 9.7-fold (90%) than the average 12.6 g tumor burden found in the untreated animals. Most excitingly, the weekly 1 mg/kg inhaled topotecan achieved a significantly better efficacy than the 2 mg/kg IV topotecan (*p* < .00001) and reduced the average tumor burden from 8.3 to 2.9 g, indicating a 5.2-fold (80%) lower tumor burden than the 2-times higher IV dose (Table 2).

**Table 2.** Inhaled topotecan is more effective in treating lung cancer than higher IV dose.

Tumors	Group	Treatments	Lung weight (g)	Tumor burden (g)	*p* Values (versus)	
			Mean ± SD	Mean ± SD	Control	IV topotecan
None	1	Naïve (*n* = 6)	1.6 ± 0.2	–	**–**	**–**
A549	2	Vehicle	14.1 ± 2.9	12.6 ± 2.7	**–**	–
	3	IV topotecan (2 mg/kg) (*n* = 15)	8.3 ± 2.3	6.7 ± 2.0	1.5E-06*	–
	4	Inhaled topotecan (1 mg/kg) (*n* = 15)	2.9 ± 0.9	1.3 ± 0.7	8.7E-11*	1.1E-07*
H1975	5	Vehicle	14.3 ± 3.5	12.8 ± 3.3		**–**
	6	IV topotecan (2 mg/kg) (*n* = 15)	15.8 ± 2.0	14.3 ± 1.8	1.6E-01	**–**
	7	Inhaled topotecan (1 mg/kg) (*n* = 15)	13.4 ± 2.7	11.9 ± 2.5	4.6E-01	1.0E-02

* Significant differences in tumor-burden between treatment groups.

**Figure 4.**
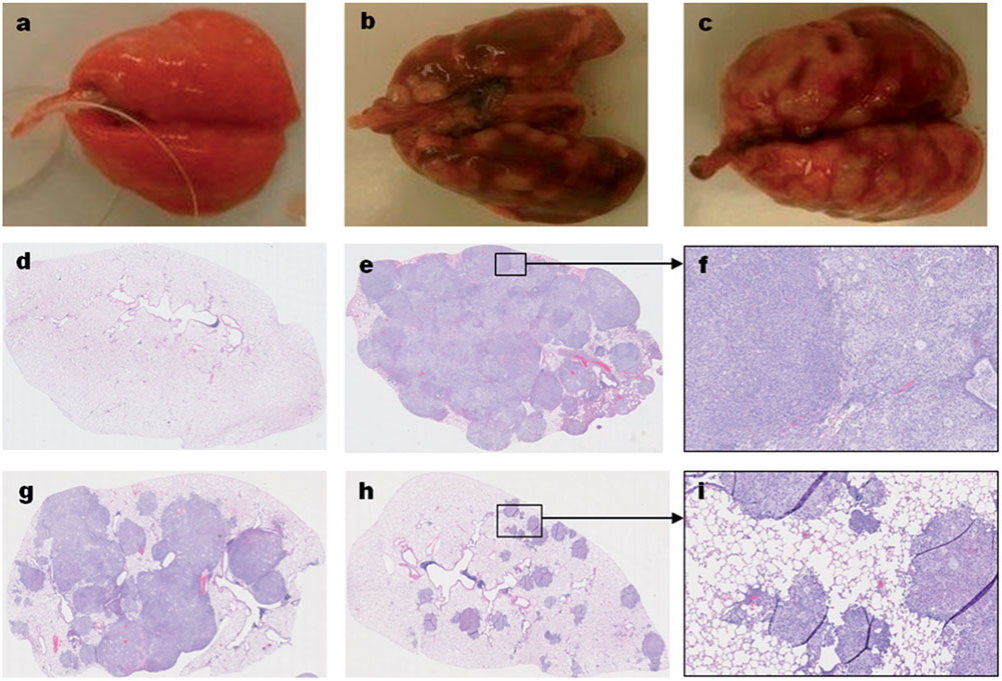
Gross and histological features of orthotopic lung tumors in rats. The gross (a–c) and microscopic (d–i) features of normal lung (a,d), A549-derived tumors (b,e–i), and H1975-derived tumors (c) are shown. Compared to the normal lungs (a) of an age-matched, cancer-free, and treatment naïve rat; the A549 (b) and H1975 (c) derived tumor-bearing lungs from untreated control animals have grossly visible, multinodular, tan-gray, masses that bulge from the pleural and visceral surfaces of the lungs, respectively. Microscopic evaluation of hematoxylin and eosin (H&E) stained sections from A549-derived tumor-bearing lungs (e–i) revealed that the lung parenchyma, smaller airways, and blood vessels are invaded by patches of cancer cells. In a vehicle-treated animal, the entire lung lobe is filled with tumor cells of mainly two types (a smaller or larger cytoplasm) and very little normal airspace is left (e,f). In a rat treated with IV topotecan, about half the lung lob is filled with patches of cancer cells that started to coalesce (g). In contrast, about 90% of the lung parenchyma of a rat treated with inhaled topotecan remained normal and the tumor nodules are much smaller and mostly noncoalescent (h,i).

The gross and microscopic features of the human lung adenocarcinoma-derived tumors in the lungs of the nude rats are shown in Figure 4. Compared to the normal lungs of age-matched control animals (Figure 4(a)), the lungs of vehicle-treated animals with either the A549-derived (Figure 4(b)) or H1975-derived (Figure 4(c)) tumors grossly show multinodular tan-gray masses that bulge from the visceral and pleural surfaces of the lungs. A549-derived tumor-bearing lungs obtained from inhaled topotecan treated animals grossly show fewer and less prominent tumor nodules than the IV topotecan and vehicle-treated animals sacrificed at the end of the study (not shown). However, the H1975-derived tumor-bearing lungs grossly showed no apparent differences between treatment groups, most likely due to the difference in growth period prior to animals becoming moribund. H&E stained normal lung and tumor-bearing lung sections from animals in the different treatment groups depicting representative low and high magnification pictures are shown in Figure 4(d–i). Detailed description of the microscopic features is included in Figure 4 legend.

### 3.5. Effects of topotecan treatment on hematological profile

The primary adverse effect of topotecan treatment in patients is hematologic toxicity mainly neutropenia and to a lesser extent thrombocytopenia and anemia (Eckardt et al., 2007). The potential toxicity of the topotecan treatments in our study was evaluated using blood and bone marrow smears as well as complete and defferential blood counts. Overall, no bone marrow suppression was observed following inhaled or IV topotecan treatments. However, a major increase in the number of neutrophils (Figure S2(A)) and total white blood cells (WBC) count (Figure S2(B)) was found in H1975-derived tumor-bearing animals (Groups 5–7) compared to the tumor-free control (Group 1) and A549-derived tumor-bearing animals (Group 2–4). Group 5 animals with untreated H1975-derived lung tumors showed the highest WBC (neutrophil) counts averaging 6-times higher WBC (neutrophil) counts than the tumor-free control animals. Treatment of H1975-derived lung cancer with IV (Group 6) or inhaled (Group 7) topotecan lowered the WBC (neutrophil) increase to 3.4 or 2.4-times than that of the tumor-free control animals, respectively. Microscopic evaluation of the H1975-derived tumors revealed large areas of necrosis that are mostly filled with neutrophils (Figure S2(C)). This indicates that the neutrophilia is likely stimulated by the H1975-derived lung tumors rather than the treatments, and its reduction in Groups 6 and 7 animals is likely related to the degree of tumor suppression achieved by the treatments.

## 4. Discussion

In this study, we have successfully manufactured a dry-powder formulation of topotecan using standard inhalation excipients. Evaluation of this novel formulation in a clinically relevant device for orally inhaled aerosols confirmed that it produces a highly respirable aerosol with high delivery efficiency under clinical and non-clinical testing. Pharmacokinetic analysis revealed that inhalation delivery of this formulation in rats increased the plasma and lung tissue half-life of topotecan and markedly increased drug exposure of lung tissue compared to the standard IV delivery. The impact of inhalation delivery in improving the efficacy of topotecan for lung cancer therapy is also demonstrated using an orthotopic lung cancer model. Inhaled topotecan significantly reduced lung tumor burden and dramatically increased survival of rats orthotopically engrafted with human lung adenocarcinoma compared to twice higher IV dose without any hematopoietic toxicity. Thus, our data support inhalation delivery of topotecan as a promising approach to improve the efficacy and expand the clinical use of this potent, but not commonly used anticancer agent for treatment of NSCLC.

Inhalation delivery of drugs for local (pulmonary) and systemic diseases has long attracted interest because it bypasses the inactivating effects of digestive and hepatic enzymes while the large surface area of the respiratory epithelium allows fast absorption (Patton & Byron, 2007). Despite these advantages, its use has been largely limited to the treatment of pulmonary diseases such as asthma and chronic obstructive pulmonary disease (COPD). However, recent advances in the development of inhalable drugs and delivery devices are re-invigorating interest about its potential use for various other diseases including lung cancer (Mangal et al., 2017). In particular, inhalation delivery of dry-powder formulations is predicted to have the best potential because of the ease of delivery and major advances in dry powder inhalers (Gagnadoux et al., 2008; Nanjwade et al., 2011; Hoppentocht et al., 2014). Dry powder inhalers offer opportunities to improve chemical and physical stability and bioavailability for some drugs and the opportunity to combine drugs into a single formulation (Hoppentocht et al., 2014; Zhou et al., 2014). In this study, we used a lab-scale spray-drying technology to manufacture a stable topotecan powder and its aerosol property was determined using the Plastiape RS01 dry powder inhaler. This inhaler is already marketed in combination with a wide range of drug products and produces efficient drug delivery in clinical trials (Elkins et al., 2014). The FDA requires that the MMAD of all orally inhaled aerosols should be 1–5 µm to achieve increased lung delivery with reduced oral deposition. The 2.9 ± 0.3 µm MMAD of our inhalable topotecan meets this requirement and was manufactured through a process that is amenable for large-scale production to support clinical testing. Inhalation of aerosols within this particle size range has been shown to result in homogeneous delivery of drugs to the lungs of clinical patients (Usmani et al., 2005; Leach et al., 2016).

We anticipated that targeted delivery of topotecan aerosol directly to the lungs through inhalation will show better drug distribution and availability in the lungs and potentially improve the half-life of topotecan in the systemic circulation. Thus, the goal of the pharmacokinetic analysis part of the study was to compare 0.7 mg/kg IV topotecan to a similar (0.7 mg/kg) and 7-times lower (0.1 mg/kg) doses of inhaled topotecan aerosol. However, as shown in Table S2, the actual dose recovered from filters in the breathing zone of the exposure system showed a slightly higher (0.79 and 0.14 mg/kg, respectively) readings than the targeted doses. Based on these results, we made the necessary adjustment for the subsequent efficacy studies and achieved the intended targets (1.00 vs. 0.99 mg/kg) as shown in the last row of Table S2.

Lung cancer is the leading cause of cancer-related mortality worldwide (Torre et al., 2015). In the US alone, approximately 243,170 new lung cancer cases and 160,420 deaths from lung cancer are expected in the year 2017 alone (Siegel et al., 2017). The immediate cause of death for approximately half of lung cancer patients is associated with extensive lung tissue damage resulting in lethal abnormalities such as pulmonary hemorrhage, pulmonary (thrombo) embolism, and infection that leads to sepsis and pneumonia (Nichols et al., 2012). Therefore, suppressing or arresting the growth of primary and locally invasive lung tumors alone by directly delivering anticancer drugs into the lungs *via* inhalation could significantly improve survival of lung cancer patients. The dramatic increase in the survival of rats with the fast-growing H1975-derived lung tumors from the control and standard IV topotecan treatment to those treated with inhaled topotecan supports this premise. The H1975 cell line is derived from a human lung adenocarcinoma and has the oncogenic EGFR tyrosine kinase activating mutation L858R as well as the T790M mutation that makes it resistant to first-generation EGFR tyrosine kinase inhibitors (TKIs) such as gefitinib and erlotinib (Kobayashi et al., 2005; Kwak et al., 2005; Pao et al., 2005). As a result, H1975-derived lung tumors are not only aggressive in terms of fast growth but are also resistant to multiple anticancer drugs including these TKIs and the front line lung cancer drug cisplatin (Tessema et al., 2012). The fact that inhaled topotecan used at half the IV dose improved survival supports evaluating this inhaled formulation as a second line for lung cancer therapy.

Further support for reevaluation of topotecan *via* the inhalation route in the clinic draws from its effects in drastically reducing the growth of the K-ras mutant A549-derived lung tumors. In a previous study, we showed that A549 lung cancer cell lines and subcutaneous xenografts derived from this cell line in mice are relatively less sensitive to *in vitro* and *in vivo* topotecan, respectively, than other more sensitive cell lines such as H1975 (Tessema et al., 2012). However, the dramatic reduction of A549-derived lung tumors achieved in this study through inhalation delivery indicates that the high level of topotecan exposure (∼30-fold higher than the 2-time higher IV dose) could effectively suppress the growth of these and potentially other less sensitive lung cancer cell types.

Lung cancer metastasis to distant sites outside of the lungs and associated complications are responsible for about half of lung cancer-related mortality (Nichols et al., 2012). The potential use of inhaled topotecan to treat metastatic lung tumors is unknown. However, the increased plasma half-life and AUC of animals treated with inhaled topotecan over a comparable dose of IV topotecan indicates that metastatic tumors likely receive higher AUC and sustained (T_1/2_) exposure to topotecan following inhalation delivery. In contrast, the maximum plasma concentration (*C*_max_) of topotecan following inhalation delivery is lower than the comparable IV dose. This suggests that the maximum topotecan level reaching metastatic cells outside of the lungs is likely lower following inhalation than IV delivery. Whether the lower *C*_max_ but higher AUC and T_1/2_ in the systemic circulation will reduce or improve the efficacy of inhaled topotecan for metastatic disease needs to be experimentally tested. Similarly, the lower plasma *C*_max_ following inhalation delivery also minimizes the maximum topotecan level reaching hematopoietic cells that are highly sensitive to the drug, thus affording the potential to lower the risk of the dose-limiting hematopoietic toxicity. Future studies are required to compare the efficacy of inhaled vs. IV topotecan on metastatic lung cancer while also defining the impact on hematopoietic toxicity. Similar efficacy of inhaled vs. IV topotecan for metastatic disease should be considered successful, if as expected, its use for lung cancer therapy is expanded due to lower toxicity. Successful use of inhaled topotecan for the treatment of lung tumors in extrapulmonary tissues will also have a wider significance as it may indicate the potential use of inhalation delivery for the treatment of malignant and nonmalignant diseases outside of the lungs.

## Supplementary Material

Supplemental Material

Supplemental Material

Supplemental Material
