# Supplementary figures and images for: Inhalation delivery of topotecan is superior to intravenous exposure for suppressing lung cancer in a preclinical model
Source: Drug Deliv. 2018 May 19;25(1):1127–36. doi: 10.1080/10717544.2018.1469688 (PMC6058531; doi:10.1080/10717544.2018.1469688)

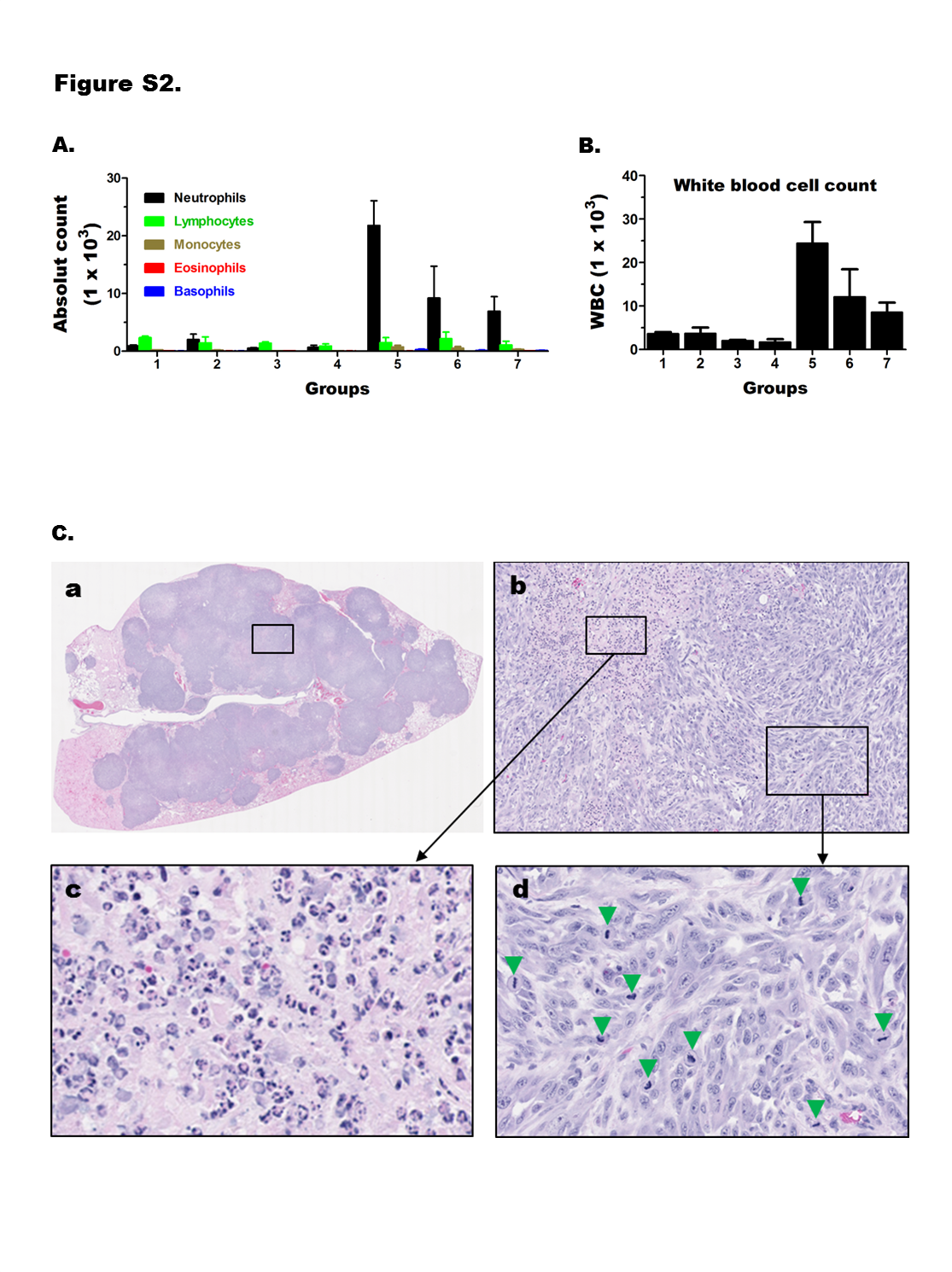

Supplement: Supplemental Material [file IDRD_A_1469688_SM3250.tif]

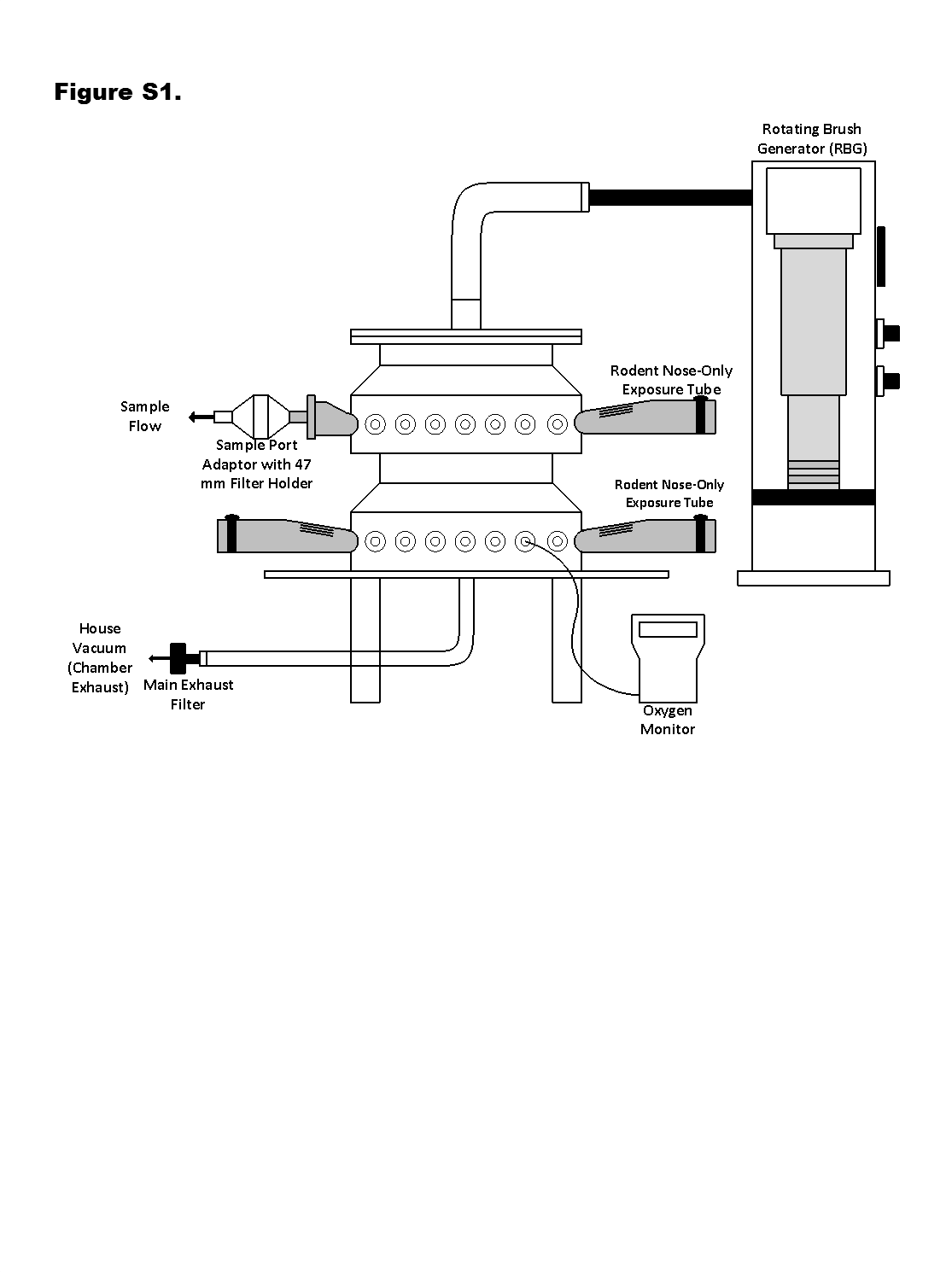

Supplement: Supplemental Material [file IDRD_A_1469688_SM3249.tif]
