# Supplementary material for: Inhalation delivery of topotecan is superior to intravenous exposure for suppressing lung cancer in a preclinical model
Source: Drug Deliv. 2018 May 19;25(1):1127–36. doi: 10.1080/10717544.2018.1469688 (PMC6058531; doi:10.1080/10717544.2018.1469688)
Supplement: Supplemental Material [file IDRD_A_1469688_SM3248.docx]

**Supplementary Tables**

**Table S1: *In Vivo* aerosol testing results of our spray-dried topotecan**

| **Formulation (w/w)** | **Trehalose/Leucine/Topotecan**  **(70/20/10)** |
| --- | --- |
| Mean mass aerodynamic diameter (MMAD, µm) | 2.9 ± 0.3 |
| Geometric standard deviation (GSD) | 1.9 ± 0.2 |
| Emitted fraction (EF, %), Capsule + Device | 94.0 ± 3.3 |
| Fine particle fraction (FPF, %), < 5 µm | 62.0 ± 1.1 |

**Table S2: Dose results for the Pharmacokinetics and efficacy studies**

| **Group** | **Target Dose (mg/kg)** | **TPT Aerosol Conc. (µg/L)** | **Actual Dose (mg/kg)** | **Particle size MMAD (GSD)** |
| --- | --- | --- | --- | --- |
| **IV** | 0.70 | NA | 0.70 | NA |
| **Low Inhalation** | 0.10 | 0.12 | 0.14 | 2.8 (1.6) µm |
| **High Inhalation** | 0.70 | 0.22 | 0.79 | 3.0 (1.7) µm |
| **Efficacy study** | 1.00 | 0.22 | 0.99 | 2.9 (1.6) µm |

**Table S3: Number of animals survived to the end of study (54 days post tumor implantation)**

| **Group** | **Lung cancer Cell line** | **Number of rats** | **End of study Survival, n (%)** | | | |
| --- | --- | --- | --- | --- | --- | --- |
|  |  |  | **None** | **Vehicle** | **2 mg/kg IV** | **1 mg/kg inhalation** |
| 1 | None * | 6 | 6 (100%) | - | - | - |
| 2 | A549 | 15 | - | 9 (60%) | - | - |
| 3 |  | 15 | - | - | 15 (100%) | - |
| 4 |  | 15 | - | - | - | 15 (100%) |
| 5 | H1975 | 15 | - | 1 (7%) | - | - |
| 6 |  | 15 | - | - | 1 (7%) | - |
| 7 |  | 15 | - | - | - | 12 (80%) |

* Animals in Group 1 serve as cancer-free and treatment-free control.

** The ‘+’ and ‘-‘ signs indicate the treatments given or not given, respectively.

**Supplementary Figure Legends**

**Figure S1:** Schematic diagram of the rodent nose-only inhalation delivery system developed and used for the delivery of the spray-dried topotecan to rats.

**Figure S2: Effect of Topotecan Treatment on the Hematological Profile of Rats. A)** Differential and **(B)** total white blood cell counts revealed that animals in Groups 5, 6, and 7 (all with H1975-derived lung tumors) have significantly higher neutrophil and total WBC counts. **C)** Microscopic evaluation of an H1975-derived lung tumor at different magnifications **(a – d)** reveals necrotic areas **(c)** that are predominantly filled with polymorphonuclear leukocytes (neutrophils) along with highly proliferating tumor cells **(d)** as shown by the large number of mitotic cells (green arrowheads).
